# Coupled Effects of the Mover Mass on Stepping Characteristics of Stick–Slip Piezoelectric Actuators †

**DOI:** 10.3390/mi17010061

**Published:** 2025-12-31

**Authors:** Zhaochen Ding, Xiaoqin Zhou, Ke Wang, Zhi Xu, Jingshi Dong, Yuqing Fan, Huadong Yu

**Affiliations:** 1 School of Mechanical and Aerospace Engineering, Jilin University, Changchun 130022, China; dingzc23@mails.jlu.edu.cn (Z.D.); xqzhou@jlu.edu.cn (X.Z.);; 2 Key Laboratory of Molecular Epigenetics, Ministry of Education, Northeast Normal University, Changchun 130024, China

**Keywords:** piezoelectric actuator, stick-slip, mover mass, stepping characteristics

## Abstract

Stick–slip piezoelectric actuators are widely used in high-precision positioning systems, yet their performance is limited by backward motion during the slip stage. Although the effects of preload force, driving voltage, and driving frequency have been extensively examined, the specific influence of mover mass and its coupling with these parameters remains insufficiently understood. This study aims to clarify the mass-dependent stepping behavior of stick–slip actuators and to provide guidance for structural design. A compact stick–slip actuator incorporating a lever-type amplification mechanism is developed. Its deformation amplification capability and structural reliability are verified through motion principle analysis, finite element simulations, and modal analysis. A theoretical model is formulated to describe the inverse dependence of backward displacement on the mover mass. Systematic experiments conducted under different mover masses, preload forces, voltages, and frequencies demonstrate that the mover mass directly affects stepping displacement and interacts with input conditions to determine motion linearity and backward-slip suppression. Light movers exhibit pronounced backward motion, whereas heavier movers improve smoothness and stepping stability, although excessive mass slows the dynamic response. These results provide quantitative insight into mass-related dynamic behavior and offer practical guidelines for optimizing the performance of stick–slip actuators in precision motion control.

## 1. Introduction

Piezoelectric actuators are widely employed in aerospace [1], biomedical engineering [2], and micro/nano-fabrication [3], owing to their high precision, fast response, and immunity to electromagnetic interference [4,5,6]. Stepping piezoelectric actuators are classified into inchworm [7], ultrasonic [8], and stick–slip types [9] according to different actuation principles. Ultrasonic actuators generate motion through high-frequency vibration of piezoelectric ceramics, offering high speed; however, they suffer from significant mechanical wear [10]. Inchworm actuators typically require the coordination of multiple piezoelectric elements. While they offer high output force and precision, their structure and control system are relatively complex [11]. In contrast, stick–slip actuators exhibit excellent performance in speed, resolution, stepping displacement, and output force, while maintaining a compact and simple structure [12]. For example, Cheng et al. developed a bionic stick–slip actuator inspired by the supporting and jumping motions of frog legs [13]. The design integrates a flexible adsorption mechanism and achieves a maximum speed of 4.23 mm/s while sustaining a vertical load of 9 N. Liu et al. presented a compact single-legged piezoelectric robot employing a parallel compliant mechanism, a compound bridge-type amplifier, and an elastic auxiliary support [14]. The robot measures 84 mm × 84 mm × 38 mm and achieves a displacement resolution of 17 nm. Yu et al. proposed a stick–slip actuator driven by an elastic energy storage approach [15]. It has a compact size of 75 mm × 60 mm × 20 mm and achieves a maximum stepping angle of 5.25 mrad and a maximum speed of 10.66 rad/s.

However, existing stick–slip actuators suffer from inherent backward motion during the slip stage, which degrades output performance, reduces efficiency and positioning accuracy, and accelerates mover wear. These effects shorten service life and reduce reliability [16,17,18]. Consequently, reducing the backward motion and improving the stepping characteristics have become major focuses in the field of stick–slip actuators. The stepping characteristics of stick–slip actuators are influenced by multiple factors, including material properties [19], contact friction [20], initial preload [21], structural stiffness [22], driving-signal parameters [23], and optimization of the flexible hinge structure [24]. For example, Wang et al. developed a model-based approach to optimize the driving signal, achieving a 29.5% improvement in speed performance compared to conventional saw-tooth signals [23]. Huang et al. reduced the normal force between the stator and mover to suppress the backward motion; however, this approach significantly decreased the output force [25]. Ding et al. designed a stick–slip actuator utilizing lever and triangular coupling amplification principles, improving the stepping performance by reducing the stiffness of the driving foot and achieving a peak displacement of 144 μm [26].

Among the factors influencing stick–slip performance, the mover mass plays a fundamental role in determining the dynamic response and overall motion stability of the actuator. Although preload force, driving voltage, and driving frequency have been widely studied [27,28], the influence of mover mass, together with its coupling with these parameters, has received little attention. Theoretically, a lighter mover has lower inertia and is more prone to reverse slipping during the slip stage, leading to larger backward displacement. Increasing the mover mass enhances inertial resistance and suppresses backward motion, thereby improving the net stepping displacement [29,30]. However, an excessively large mover mass reduces energy efficiency, degrades dynamic response, and increases overall actuator size [31]. In practical stick–slip actuators, the effect of mover mass does not act in isolation but interacts with other system parameters. It interacts with the preload at the frictional interface, influencing the transition between sticking and slipping. In addition, the mover mass couples with driving-signal parameters, such as voltage and frequency, jointly shaping the balance between forward displacement and backward motion within each actuation cycle. These coupled effects have a decisive influence on stepping efficiency, motion linearity, and stability. Despite their importance, systematic studies on these mass-related coupling mechanisms are still lacking.

To guide structural design, a systematic investigation into the influence of mover mass on the stepping characteristics is carried out in this study. First, a typical stick–slip actuator structure is modeled and optimized using finite element analysis to demonstrate the feasibility of the proposed approach. Subsequently, a theoretical model is developed to describe the relationship between the mover mass and backward displacement. Finally, experiments under different mover masses, initial preloads, driving voltages, and driving frequencies are conducted to validate the theoretical model and offer practical guidelines for improving stick–slip actuator design and performance.

## 2. Hinge Design and Analysis

### 2.1. Design and Motion Principle of the Actuator

**Figure 1:**
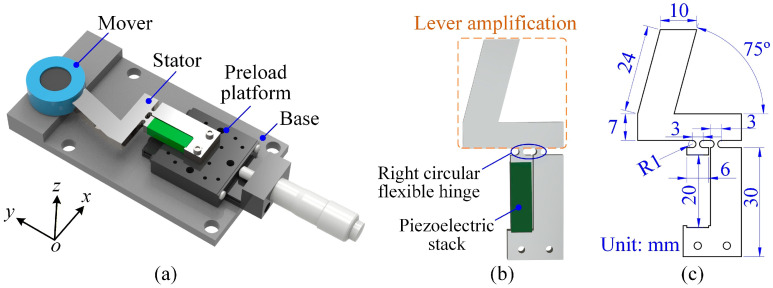
(**a**) Three-dimensional model of a typical stick–slip piezoelectric actuator. (**b**) Configuration of the stator. (**c**) Detailed dimensions of the flexible hinge mechanism.

To investigate how the mover mass affects the actuator’s stepping characteristics, a typical stick–slip actuator was designed (see Figure 1a). The actuator system comprises a mover, a preload platform, a base, and a stator. A rolling bearing supports the mover, with its inner ring fixed to the base and its outer ring in contact with the stator. The initial preload force between the stator and mover can be adjusted through the preload platform. As illustrated in Figure 1b, the stator mainly consists of a piezoelectric stack and a flexible mechanism incorporating a lever-type displacement amplification structure. The upper branch of the flexible hinge mechanism acts as a mechanical lever, amplifying the small elongation of the piezoelectric stack and transmitting it to the output contact surface. At the rotation point of the lever, the right circular flexible hinges are adopted. Such flexible hinges provide smooth rotational compliance with reduced stress concentration, which ensures stable deformation and improves fatigue resistance during high-cycle actuation.

The detailed dimensions of the flexible hinge mechanism are shown in Figure 1c. The fixed end of the stator is designed according to the size of the piezoelectric stack. The dimensions of the lever amplification section are selected to balance displacement amplification and structural compactness. In addition, a 75° inclination angle is intentionally introduced so that the flexible hinge mechanism forms a parasitic-motion structure. This configuration enables the output tip to generate both a forward driving force along the *x*-direction and a preload component along the *y*-direction when contacting the mover, thereby promoting stable stick–slip motion.

**Figure 2:**
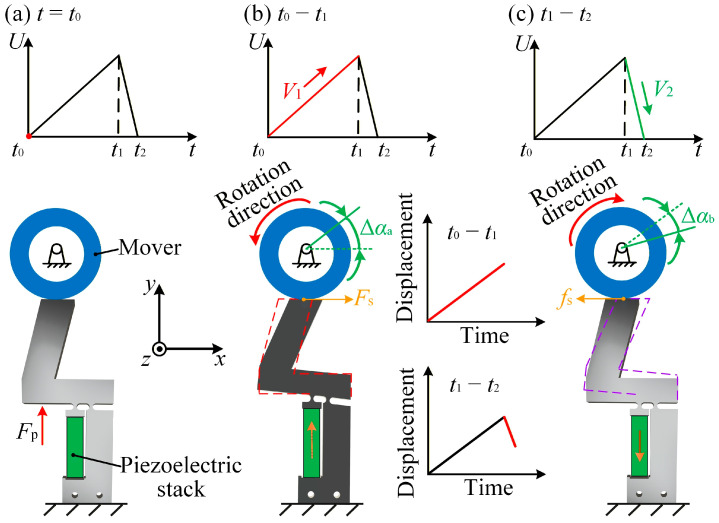
Motion principle of the stick–slip actuator. (**a**) Initial state with no voltage input. (**b**) Voltage gradually increases during the stick stage. (**c**) Voltage rapidly decreases during the slip stage.

The motion principle of the actuator consists of three stages:

(a) Initial stage: As shown in Figure 2a, at time *t* = *t*_0_, the preload platform knob is adjusted to establish elastic contact between the flexible hinge and mover. This adjustment ensures an appropriate preload force (*F*_p_), which is essential for stable operation in subsequent motion cycles.
(b) Stick stage: Between *t*_0_ and *t*_1_, the driving voltage increases slowly, causing the piezoelectric stack to elongate and deform the flexible hinge (see Figure 2b). Due to the static friction force (*F*_s_) at the interface between the flexible hinge and mover, the mover rotates counterclockwise, producing a forward angular displacement (Δ*α*_a_) during the stick stage. The effectiveness of this stage is primarily determined by the magnitude of static friction and displacement amplification provided by the flexible hinge structure.
(c) Slip stage: Between *t*_1_ and *t*_2_, the driving voltage decreases rapidly, causing the piezoelectric stack to contract and the flexible hinge to reset quickly, as illustrated in Figure 2c. Driven by the sliding friction force (*f*_s_), the mover rotates clockwise by an angular displacement (Δ*α*_b_), representing the backward displacement in the slip stage. This backward motion partially offsets the forward progress generated during the previous stick stage.

The stepping displacement (Δ*α*) for each motion cycle is defined as Δ*α* = Δ*α*_a_ − Δ*α*_b_. The forward displacement (Δ*α*_a_) is greater than the backward displacement (Δ*α*_b_), resulting in a net forward step after each motion cycle. The stick–slip motion is enabled by the asymmetric loading and unloading of the piezoelectric stack: a slow extension allows static friction to dominate, whereas a rapid contraction breaks the sticking condition and produces a smaller backward motion. This asymmetry is the essential mechanism that generates a net forward displacement. Increasing the preload enhances the normal force and frictional coupling, increasing the driving voltage enlarges the deformation of the flexible hinge, and increasing the driving frequency shortens the slip duration. These factors jointly tune the balance between forward sticking displacement and backward sliding displacement.

### 2.2. Finite Element Analysis

To optimize the output performance of the designed actuator, the flexible hinge mechanism was modeled and simulated with the commercial finite element software ANSYS Workbench Student Version 2021 R2 prior to experimental validation. The flexible hinge was fabricated from 65Mn spring steel, with a Young’s modulus of 1.96 × 10^11^ N/m^2^, a Poisson’s ratio of 0.30, a density of 7810 kg/m^3^, and a yield strength of 784 MPa. The piezoelectric stack (AE0505D16F, Tokin, Japan) has a size of 5 mm × 5 mm × 20 mm, with a maximum driving voltage of 150 V and an equivalent stiffness of 48.9 × 10^6^ N/m.

To verify the numerical accuracy of the finite element analysis, a mesh convergence study was performed by comparing simulation results obtained with different mesh sizes. A spatial eight-node hexahedral element was adopted for discretization. Considering that the minimum characteristic dimension in the transition region of the right circular flexible hinge is approximately 1 mm, the maximum mesh size was initially set to 1 mm. As summarized in Table 1, with decreasing mesh size, the output displacement in the *x*-direction varies only within a small range, and the difference in amplification ratio remains within 0.01. When the mesh size is further reduced below 0.3 mm, both the output displacement and amplification ratio remain unchanged, indicating that the numerical results have reached convergence. Therefore, a mesh size of 0.3 mm was selected for the finite element analysis.

**Figure 3:**
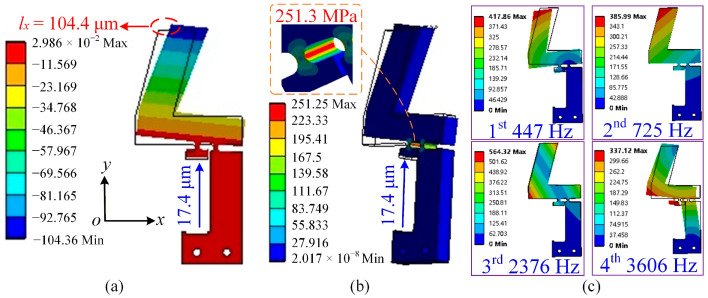
Finite element simulation results. (**a**) Total deformation corresponding to an input displacement of 17.4 μm. (**b**) Equivalent stress distribution corresponding to an input displacement of 17.4 μm. (**c**) First four mode shapes of the flexible hinge mechanism, with natural frequencies of 447 Hz, 725 Hz, 2376 Hz, and 3606 Hz, respectively.

To simulate actual operating conditions, a fixed constraint was applied at the bottom of the flexible hinge mechanism. Meanwhile, the output end of the lever amplification mechanism was constrained in the *y*-direction and allowed to move only along the *x*-direction. As shown in Figure 3a, a displacement load of 17.4 μm was applied at the input end of the flexible hinge. The resulting output displacement along the *x*-axis reached 104.4 μm, corresponding to an amplification ratio of 6.00. Additionally, to validate the safety of the designed flexible hinge, a stress analysis was performed. When a displacement load of 17.4 μm was applied, the maximum equivalent stress at the right circular flexible hinge reached 251.3 MPa, which is well below the yield strength of 65Mn spring steel (784 MPa). This indicates that the designed flexible hinge can operate effectively and safely over an extended period (see Figure 3b).

In addition, a modal analysis was carried out to identify the natural frequencies of the flexible hinge mechanism. The first four natural frequencies are 447 Hz, 725 Hz, 2376 Hz, and 3606 Hz, as shown in Figure 3c. Since the stick–slip actuator relies on quasi-static loading rather than dynamic resonance, the driving frequency must remain below the first natural frequency to avoid resonance-induced vibration. Therefore, the modal analysis results are used as a design verification criterion to define a safe frequency range. In this study, the maximum driving frequency is limited to 80 Hz, corresponding to approximately 18% of the first natural frequency. This safety margin avoids excessive deformation, unstable contact forces, and vibration-induced disturbances, thereby ensuring stable and repeatable stepping characteristics.

### 2.3. The Influence of Mover Mass on the Backward Displacement

To evaluate the effect of mover mass on the backward displacement during the slip stage, the dynamic response of the actuator under a single-stator stick–slip drive is considered. During the slip stage, the mover experiences a sliding friction force in the opposite direction of its forward motion. According to the momentum theorem [29,32], the relationship between the sliding friction force (*f*_s_) and speed variation (Δ*v*_slip_) of the mover is:

(1) $$f_{s}\Delta t_{\mathrm{slip}}=m_{r}\Delta v_{\mathrm{slip}}$$

where *m*_r_ is the mover mass, and Δ*t*_slip_ is the duration of the slip stage.

The speed variation (Δ*v*_slip_) is related to the backward displacement (Δ*α*_b_), as follows:

(2) $$\Delta v_{\mathrm{slip}}=\frac{\Delta \alpha_{b}r_{m}}{\Delta t_{\mathrm{slip}}}$$

where *r*_m_ represents the radius of rotation.

Equation (2) is substituted into (1), and the result is obtained as

(3) $$\Delta \alpha_{b}=\frac{f_{s}\cdot r_{m}\Delta t_{\mathrm{slip}}^{2}}{J_{r}}=\frac{f_{s}\Delta t_{\mathrm{slip}}^{2}}{m_{r}r_{m}}$$

This equation indicates that the backward angular displacement is inversely proportional to the mover mass. Physically, a larger mover mass results in a greater moment of inertia, which helps suppress the backward motion during the slip stage.

It should be noted that the above theoretical analysis is based on several simplifying assumptions. In typical stick–slip piezoelectric actuators, a saw-tooth driving signal with a duty cycle close to 100% is commonly adopted to reduce the influence of the slip stage. As a result, the duration of the slip stage (Δ*t*_slip_) is relatively short. Under this condition, the sliding friction force can be reasonably approximated as constant during a single slip event. In addition, the normal contact force is assumed to be dominated by the applied preload. For stick–slip actuators capable of providing stable output force, the gravitational force of the mover is typically much smaller than the applied preload force [33,34]. Therefore, variations in mover mass mainly influence the inertial response of the system rather than the magnitude of the sliding friction force.

Furthermore, the present model focuses on the backward displacement behavior under low-frequency operating conditions, where the stick–slip effect is more pronounced. With increasing driving frequency, the mover exhibits higher momentum and tends to maintain its motion state, thereby suppressing the backward motion during the slip stage [35]. To highlight the dominant influence of mover mass, secondary effects such as surface wear, temperature rise, and high-frequency vibration are not considered in this model.

Under these assumptions, increasing the mover mass reduces backward displacement and improves stepping stability. However, an excessively large mover mass may lead to a slower dynamic response, reduced adaptability at high frequencies, and an increased actuator size. Therefore, the mover mass should be selected to balance motion stability and actuation performance.

## 3. Experiments and Analysis

### 3.1. Experimental System

**Figure 4:**
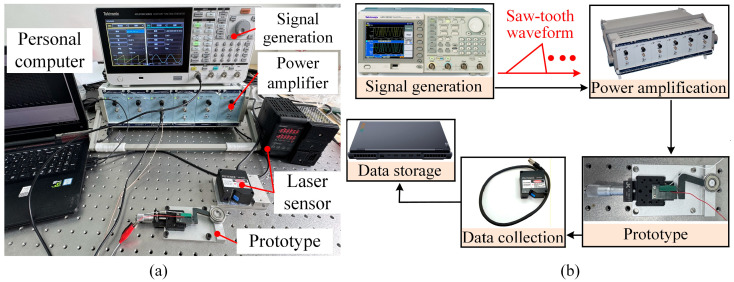
(**a**) Experimental system and prototype. (**b**) The corresponding control process.

To maintain the reliability and precision of the test results, all experiments were carried out under controlled conditions. As shown in Figure 4a, the experimental platform consisted of five main components: the actuator prototype, a laser displacement sensor (LK-H020, KEYENCE, Osaka, Japan), a function generator (AFG3022C, Tektronix, Beaverton, OR, USA), a power amplifier (E00A6, Core Morrow, Harbin, China), and a personal computer for data processing. The entire setup was placed on an air-floating vibration isolation platform to minimize the influence of environmental vibrations, thereby reducing measurement uncertainty caused by external disturbances. The displacement of the mover was measured using the laser displacement sensor with a resolution of 10 nm. The sampling frequency was set to 20 kHz, which is sufficient to capture the actuator motion at the maximum driving frequency used in this study. The displacement signals were recorded at this high sampling rate and directly used for subsequent data analysis without additional digital filtering. A precision stage was used for alignment to ensure that the sensor measured along the correct axis. All experiments were conducted under normal laboratory conditions at room temperature (approximately 23 ± 1 °C) and moderate relative humidity.

During the experiment, a saw-tooth driving waveform with a high duty cycle was generated by the function generator, as shown in Figure 4b. The slow voltage ramp allows the piezoelectric stack to extend gradually, promoting a stable stick stage and steady driving of the mover. In contrast, the rapid voltage drop enables fast unloading, reducing the influence of the slip stage and suppressing the backward motion. The driving signal was amplified by the power amplifier and applied to the stator. The flexible hinge was fabricated from 65Mn spring steel and heat-treated by quenching to enhance hardness and elastic stability. No lubrication or surface coating was applied at the stator-mover contact interface, which can therefore be regarded as a dry friction contact under controlled preload conditions. The resulting displacement was continuously measured using the laser displacement sensor. The sensor offered high-resolution measurements, enabling precise tracking of the actuator response. The displacement data were sent to the personal computer, where they were recorded and processed using analysis software. This control workflow ensured tight synchronization and accurate capture of the driving signal, actuator response, and sensor output throughout the testing process.

### 3.2. Coupled Influence of Mover Mass and Preload on Stepping Characteristics

**Figure 5:**
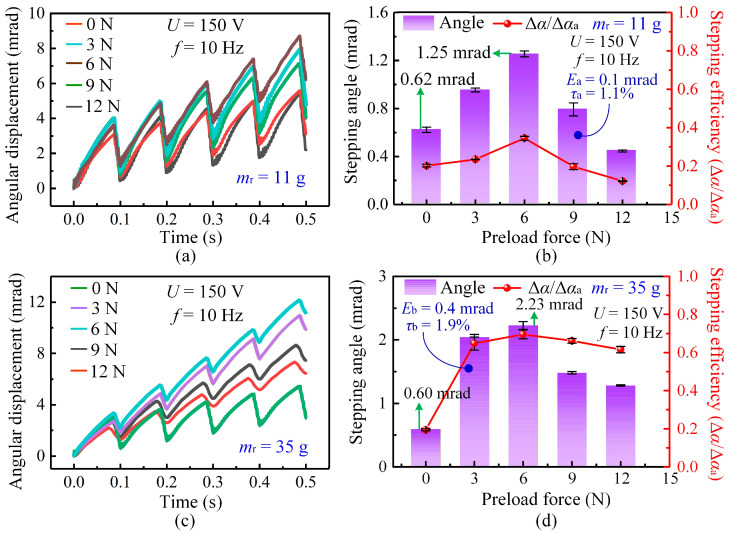
Influence of preload force and mover mass on the stepping characteristics of the actuator at 150 V and 10 Hz. (**a**) Angular displacement curves under different preload forces for the 11 g mover. (**b**) Variation in stepping angle and stepping efficiency with preload force for the 11 g mover. (**c**) Angular displacement curves under different preload forces for the 35 g mover. (**d**) Variation in stepping angle and stepping efficiency with preload force for the 35 g mover.

To clarify the coupled influence of mover mass and preload force on the stepping characteristics, experiments were conducted under various combinations of these parameters. When the mover mass is 11 g, Figure 5a shows pronounced backward motion in each cycle. The low inertia of the light mover fails to resist the rapid reversal of the stator during the slip stage, resulting in a reduced net stepping angle. As the preload increases, the stepping angle initially increases and then decreases. At low preload levels, insufficient contact force leads to partial slip and energy loss. A moderate preload enhances contact stiffness and frictional coupling, thereby producing the maximum stepping angle. When the preload becomes excessive, increased friction suppresses stator deformation and reduces the output.

As shown in Figure 5b, stepping efficiency (*η*) is defined as the ratio of the net stepping angle per cycle (Δ*α*) to the forward displacement (Δ*α*_a_) generated during the stick stage, i.e., *η* = Δ*α*/Δ*α*_a_. This definition is intended to quantify the extent of backward motion within each stick–slip cycle. A higher stepping efficiency indicates a reduction in backward displacement and more effective utilization of the forward motion generated during the stick stage. It should be emphasized that stepping efficiency is a kinematic performance metric rather than an energy-based efficiency. Unlike energy efficiency, which is generally defined as the ratio of mechanical output energy to electrical input energy, stepping efficiency focuses on motion effectiveness and the suppression of backward slip. This metric is particularly suitable for stick–slip piezoelectric actuators, in which backward motion is inherent in each cycle and strongly affects positioning accuracy. Moreover, stepping efficiency can be directly obtained from displacement measurements, making it an intuitive and convenient metric for comparing the stepping characteristics among different stick–slip actuators without requiring detailed energy measurements. For all stepping-angle and stepping-efficiency results presented in Figure 5, each data point represents the average of three independent experiments conducted under identical operating conditions. For the 11 g mover, the stepping efficiency reaches its peak at approximately 6 N, confirming this preload as the optimal value.

When the mover mass increases to 35 g, Figure 5c shows that backward motion is effectively suppressed. The larger inertia helps the mover maintain its motion direction during the rapid contraction of the piezoelectric stack, thereby improving stepping stability and linearity. As shown in Figure 5d, both the stepping angle and stepping efficiency increase as the mover mass rises from 11 g to 35 g. The maximum average stepping angle increases from approximately 1.25 mrad to 2.23 mrad, accompanied by an improvement in stepping efficiency. Across the tested preload conditions, the maximum deviation (*E*_b_) of the stepping angle remains below 0.4 mrad, and the mean relative error (*τ*_b_) of the stepping efficiency is within 2%, indicating good experimental repeatability. Notably, the optimal preload remains nearly constant at about 6 N for both mover masses. This consistency indicates that the optimal preload is mainly determined by the geometric configuration and contact stiffness at the stator-mover interface, making it an inherent characteristic of the fabricated actuator. Therefore, subsequent experiments on mover-mass effects were conducted under this fixed optimal preload.

It is also worth noting that the 35 g mover exhibits a smaller initial stepping angle (0.60 mrad) than the 11 g mover (0.62 mrad) under zero preload. This behavior occurs because the heavier mover requires a larger initial driving force to overcome static friction; under insufficient preload, the available driving energy is inadequate to initiate motion. Consequently, the lighter mover is easier to start under such conditions.

Overall, these results demonstrate that mover mass and preload jointly govern the stepping characteristics of the actuator. A smaller mover mass intensifies backward motion due to lower inertia, whereas a larger mover mass enhances stepping stability but requires a higher force to initiate movement. The nearly constant optimal preload further indicates that the actuator structural design dictates the preferred contact state, providing a reliable basis for evaluating mover mass effects under operational conditions.

### 3.3. Coupled Influence of Mover Mass and Voltage on Stepping Characteristics

**Figure 6:**
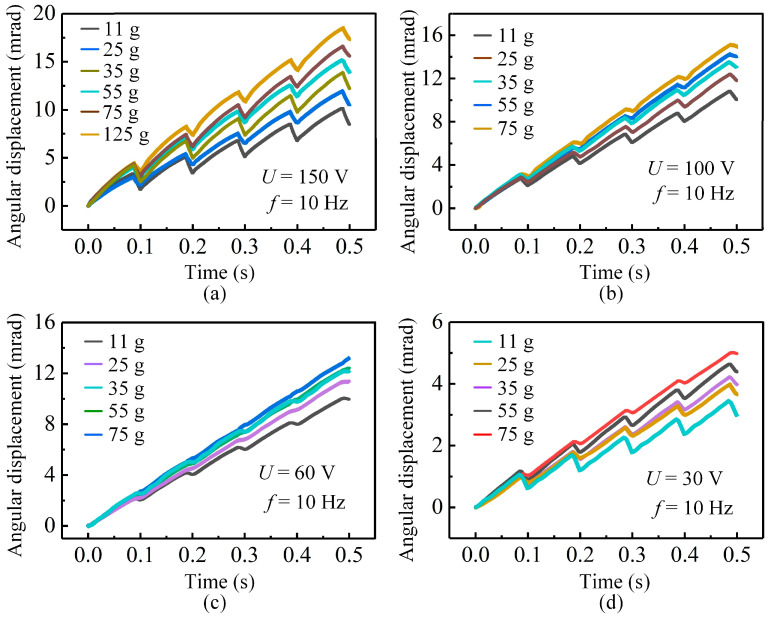
Stepping displacements of the actuator under different driving voltages for various mover masses: (**a**) 150 V; (**b**) 100 V; (**c**) 60 V; (**d**) 30 V.

The driving voltage strongly affects the stepping characteristics of the actuator by changing both the deformation of the piezoelectric stack and the frictional interaction between the stator and mover. As shown in Figure 6a, at a high voltage of 150 V, the actuator produces a large step amplitude but also exhibits clear backward motion in each cycle. As the mover mass increases, the backward motion is reduced, because the larger inertia enables the mover to resist the rapid return of the stator during the slip stage. For example, when the mover mass reaches 125 g, the maximum stepping angle increases to 3.46 mrad with noticeably improved stability.

When the voltage is reduced to 100 V, the step amplitude decreases, but the displacement curves become much smoother for all mover masses, as depicted in Figure 6b. The smaller deformation of the piezoelectric stack reduces variation in the normal force, which in turn lowers sliding friction and suppresses backward motion. Although the forward displacement in each cycle is smaller than that at 150 V, the reduced disturbance in both the stick and slip stages produces more continuous and stable motion.

Further reducing the voltage to 60 V leads to an even smaller step amplitude, but the displacement curves for different mover masses become highly consistent, as displayed in Figure 6c. In this voltage range, the reduced actuation stroke decreases both the forward driving force and the reverse frictional disturbance. As a result, the output motion becomes smoother and nearly linear. For instance, the 75 g mover achieves a maximum stepping angle of 1.84 mrad with excellent continuity, indicating that 60 V is suitable for long-term and efficient operation. However, when the voltage is further reduced to 30 V, the elongation of the piezoelectric stack becomes limited, resulting in insufficient deformation of the flexible hinge mechanism and a degradation of the stick–slip characteristics (see Figure 6d). Consequently, the effective driving displacement and force transmitted to the contact interface are reduced, leading to the reappearance of backward motion and degraded stepping performance. This indicates that 30 V corresponds to a low-voltage operating regime with degraded stepping characteristics rather than a no-motion condition.

**Figure 7:**
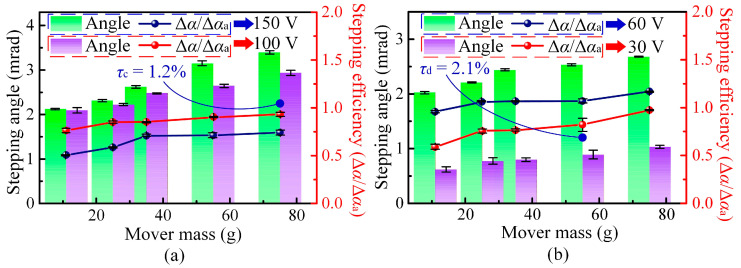
Influence of mover mass on stepping performance under different driving voltages. (**a**) Stepping angle and stepping efficiency at 150 V and 100 V. (**b**) Stepping angle and stepping efficiency at 60 V and 30 V.

Figure 7 further summarizes the influence of mover mass on the stepping angle and stepping efficiency at different driving voltages. For all tested voltages, the net stepping angle increases monotonically with mover mass, confirming that a larger inertia consistently suppresses the backward motion and enhances the effective stepping angle. However, the trend of stepping efficiency differs from that of the stepping angle. At a given mover mass, the stepping efficiency at 100 V is higher than that at 150 V (see Figure 7a), while the efficiency at 60 V exceeds that at 30 V (see Figure 7b). This means that an excessively high or low driving voltage is not beneficial for efficient operation: a high voltage amplifies both forward angle and backward slip, while a low voltage leads to insufficient stick–slip action. Therefore, a moderate driving voltage combined with an appropriately increased mover mass achieves a better balance between a large stepping angle and high stepping efficiency, in good agreement with the theoretical predictions in Section 2.3. It should be noted that although the net stepping angle increases with mover mass, the stepping efficiency only shows a slight improvement and tends to saturate. As the mover mass increases, the backward angle (Δ*α*_b_) decreases approximately in inverse proportion to the mass, whereas the forward angle (Δ*α*_a_) changes only slightly. When the driving voltage is 100 V, the mean relative error (*τ*_c_) of the stepping efficiency is approximately 1.2% for a mover mass of 75 g. In contrast, at a lower driving voltage of 30 V, the corresponding error (*τ*_d_) increases to about 2.1% for a mover mass of 55 g.

### 3.4. Coupled Influence of Mover Mass and Frequency on Stepping Characteristics

**Figure 8:**
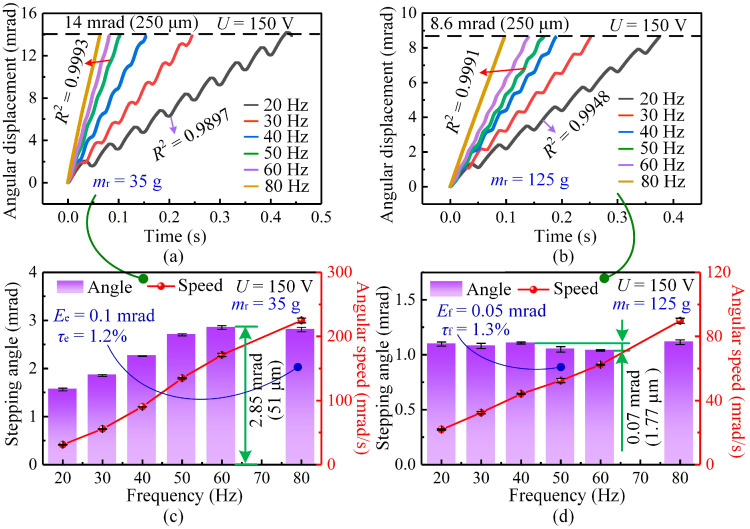
Variation in stepping characteristics with driving frequency under a driving voltage of 150 V. (**a**) Angular displacement curves at different frequencies for a mover mass of 35 g. (**b**) Angular displacement curves at different frequencies for a mover mass of 125 g. (**c**) Influence of driving frequency on the stepping angle and angular speed for a 35 g mover. (**d**) Influence of driving frequency on the stepping angle and angular speed for a 125 g mover.

The effect of driving frequency on the stepping characteristics of the actuator is investigated, as shown in Figure 8a. When the driving frequency is 20 Hz and the mover mass is 35 g, the displacement curve shows noticeable oscillations and backward motion in each cycle, resulting in relatively low linearity (*R*^2^ = 0.9897). These fluctuations arise from the lower kinetic energy and reduced inertia, which make the mover more susceptible to reverse slipping during the slip stage. When the frequency increases, both the stick and slip stages become more consistent: the slip duration becomes shorter, the backward displacement is reduced, and the motion evolves into a nearly uniform stepping process. At driving frequencies of 50 Hz and above, the displacement curves display almost perfect linearity (*R*^2^ = 0.9993), indicating that the system reaches a stable and repeatable operating regime.

When the mover mass is increased to 125 g at the same 20 Hz frequency, the curve becomes smoother and the linearity improves to *R*^2^ = 0.9948 (see Figure 8b). The added inertia helps suppress the backward motion, contributing to improved motion consistency. As the driving frequency increases to 50 Hz and above, the stepping performance is significantly enhanced for both 35 g and 125 g movers. At 50 Hz, the displacement curves for both masses display near-perfect linearity (*R*^2^ = 0.9991), indicating that higher driving frequency increases the average speed and momentum of the mover, thereby improving overall smoothness.

Figure 8c,d further summarize the variations in stepping angle and angular speed with driving frequency. For the 35 g mover, the angular speed increases steadily from 20 Hz to 80 Hz, and the stepping angle increases from 20 Hz to 60 Hz, reaching a maximum of 2.85 mrad (equivalent to 51 μm). When the frequency is 80 Hz, the maximum deviation (*E*_e_) of the stepping angle is approximately 0.1 mrad, and the mean relative error (*τ*_e_) of the stepping efficiency is about 1.2%. For the heavy mover of 125 g, the angular speed still increases with frequency, but the stepping angle varies only within a narrow range, with a maximum difference of 0.07 mrad (1.77 μm). At 50 Hz, the corresponding maximum deviation (*E*_f_) and mean relative error (*τ*_f_) are approximately 0.05 mrad and 1.3%, respectively. Since the backward angle is already sufficiently small for this mass, increasing the driving frequency has a limited effect on the net stepping angle. Therefore, for heavy movers, increasing the driving frequency mainly enhances speed but does not significantly change the stepping angle.

Overall, these results show that driving frequency and mover mass jointly determine the stepping characteristics of the actuator. Light movers are more sensitive to frequency variation, which effectively improves both motion linearity and stepping angle. In contrast, heavy movers provide sufficient inertia to suppress the backward motion, making frequency primarily affect operating speed rather than step amplitude.

## 4. Discussion: Merits, Limitations, and Application Considerations

The proposed stick–slip piezoelectric actuator adopts a simple and representative structure consisting of a single piezoelectric stack and a lever-based flexible amplification mechanism. A major advantage of this design is its structural simplicity and the use of a motion principle consistent with a typical stick–slip driving process. By employing a conventional and well-understood actuation scheme, the present study provides a suitable experimental platform for investigating the coupled effects of mover mass on stepping characteristics. As a result, the derived trends and conclusions are broadly applicable rather than specific to a highly customized structure.

Another merit of the proposed design lies in the use of a relatively rigid output structure on the stator side, which increases the equivalent stiffness of the actuation system. The enhanced stiffness contributes to more stable contact conditions between the stator and mover, enables a wider and more controllable preload range, and improves the consistency of force transmission during the stick stage. These features are particularly beneficial for systematic parametric studies, such as the investigation of mover-mass-dependent stepping behavior conducted in this study.

Nevertheless, the proposed design involves several trade-offs. The increased structural stiffness and the use of a flexible hinge operating within a limited deformation range result in a reduced amplification ratio, which constrains the forward displacement generated during the stick stage. In addition, the higher contact forces associated with increased stiffness may lead to larger sliding friction during the slip stage, making backward motion more pronounced under certain operating conditions. While this backward motion represents a limitation in terms of motion efficiency, it also provides a clearer experimental basis for observing and quantifying the influence of mover mass on stepping behavior, thereby facilitating the identification of coupled trends.

From an application-oriented perspective, the results of this study provide practical guidance for selecting mover mass and designing stick–slip actuators under different load conditions. Within an appropriate operating range, a relatively larger mover mass is beneficial for applications requiring high motion stability and reduced backward displacement, such as optical positioning and precision alignment. In contrast, a smaller mover mass may be preferred for micro-assembly tasks that demand faster dynamic response, although this typically involves increased sensitivity to backward motion. In practice, the mover mass should be selected in accordance with the expected load conditions so that the actuator operates near an optimal stepping region rather than at extreme mass or load levels.

Furthermore, although long-term effects such as wear and temperature rise were not experimentally investigated in this study, they may affect stepping performance through gradual changes in contact conditions during prolonged operation. Accordingly, future work will focus on extended cycling tests, contact wear characterization, and temperature monitoring to further assess the long-term stability and scalability of the proposed actuator.

## 5. Conclusions

In summary, a typical stick–slip piezoelectric actuator was developed to investigate the influence of mover mass on stepping characteristics. A theoretical model describing the inverse dependence of backward displacement on mover mass was established based on momentum considerations during the slip stage, and finite element simulations verified the structural safety and displacement amplification capability. Experiments conducted under different mover masses, preload forces, driving voltages, and frequencies lead to the following conclusions:

(1) Increasing the mover mass suppresses backward displacement and improves motion linearity due to increased inertia. This behavior represents a general physical trend that is applicable to stick–slip actuators operating under low-frequency, non-resonant conditions, although excessive mass may slow the dynamic response.
(2) Higher driving frequencies enhance the mover momentum and reduce backward slip, especially for heavier movers. This effect reflects a general characteristic of stick–slip actuation but is quantitatively influenced by the specific structural and dynamic properties of the actuator.
(3) For the proposed actuator design, an initial preload of approximately 6 N provides optimal frictional engagement, and a moderate driving voltage (around 60 V) yields stable stepping with minimal fluctuation. These values should be interpreted as design-dependent results rather than universal operating conditions.

Overall, mover mass determines not only the intrinsic dynamic behavior of the actuator but also its sensitivity to preload and driving-signal parameters. These findings offer practical guidelines for selecting the mover mass and operating conditions to achieve stable and precise motion in compact stick–slip piezoelectric actuators.

## Figures and Tables

**Table 1:** Results of the mesh convergence analysis.

Mesh Size	*x*-Direction Output Displacement	Amplification Ratio
1.0 mm	104.21 μm	5.99
0.8 mm	104.24 μm	5.99
0.5 mm	104.32 μm	6.00
0.3 mm	104.38 μm	6.00
0.1 mm	104.38 μm	6.00

## Data Availability

The original contributions presented in this study are included in the article. Further inquiries can be directed to the corresponding author.
